# Crystal structure or chemical composition of salt–sugar-based metal–organic frameworks: what are the nonlinear optical properties due to?

**DOI:** 10.1107/S2052520621004637

**Published:** 2021-07-06

**Authors:** Domenica Marabello, Paola Antoniotti, Paola Benzi, Fabio Beccari, Carlo Canepa, Elena Cariati, Alma Cioci, Leonardo Lo Presti

**Affiliations:** a Dipartimento di Chimica, University of Torino, Via P. Giuria 7, Torino 10125, Italy; b CrisDi – Interdepartmental Center for Crystallography, University of Torino, Via P. Giuria 7, Torino 10125, Italy; c Dipartimento di Chimica, Università degli Studi di Milano, Milano, Italy; d INFN – Laboratori Nazionali di Frascati, Frascati, Italy

**Keywords:** metal–organic frameworks, nonlinear optical properties, second-harmonic generation efficiency, isomorphous compounds, *ab initio* calculations, structure–property relationships

## Abstract

In the last few decades optical imaging techniques based on nonlinear optical properties have been of interest for biosensing applications. This work focuses on two isostructural and isomorphous sugar-derived metal–organic frameworks (MOFs) with second-harmonic generation (SHG) properties, in order to investigate their possible application as biosensors in view of their high biocompatibility. Combining 2-de­oxy-d-galactose with the metal halogenides Ca*X*_2_ (*X* = Br, I), two new isomorphous MOFs of formula [Ca(C_6_H_12_O_5_)_2_]*X*_2_ were obtained and characterized through single-crystal X-ray diffraction. The first-order static hyperpolarizability and second-order susceptibility were estimated by *in vacuo* and in-crystal density functional theory calculations, and compared with the experimental SHG response of powdered samples. The parameters influencing the SHG response of these compounds were investigated by comparison with similar previously analysed MOFs, to understand how to design more efficient materials to be used as nanoprobes by exploiting crystal engineering techniques.

## Abstract 2

Two new isomorphous metal–organic frameworks of formula [Ca(C_6_H_12_O_5_)_2_]*X*_2_ have been synthesized and the parameters influencing their nonlinear optical response investigated.

## 1. Introduction   

In the last few decades optical imaging techniques based on the nonlinear optical (NLO) properties, and in particular the second-harmonic generation (SHG), of materials have been of interest for biosensing applications (Boyd, 2003 ▸), *i.e.* these materials can be used in biological systems for the selective detection of biostructures. The advantage of SHG-based nanoprobes is that in principle they do not bleach or blink, and the second-harmonic signal does not saturate with increasing illumination intensity (Campagnola & Loew, 2003 ▸; Dempsey *et al.*, 2012 ▸; Huang *et al.*, 2006 ▸; Jin, 2012 ▸; Liu *et al.*, 2017 ▸; McKinlay *et al.*, 2010 ▸; Pantazis *et al.*, 2010 ▸; Park, 2009 ▸). SHG nanoprobes are often composed of inorganic compounds or metals that cannot be considered as biocompatible materials (Holzinger *et al.*, 2014 ▸). For many years, our research has been focused on sugar-derived metal–organic frameworks (MOFs) with SHG properties and their potential application as biosensors in view of their high biocompatibility. Our main interest is related to the parameters influencing the SHG response of this type of compound, and to be able to design more efficient materials by exploiting crystal engineering techniques.

It is well known that a lack of inversion symmetry in a crystal structure is necessary to generate an SHG response. We thus focused our attention towards sugar-based MOFs that are intrinsically not centrosymmetric. Our previous work on this subject was aimed at investigating the influence of composition on the SHG response, analysing four iso­morphous MOFs based on β-d-fructose and alkali earth halogenides, *MX*_2_ (*M* = Ca, Sr; *X* = Cl, Br) (Marabello *et al.*, 2017 ▸). We showed that the cation did not play a significant role, while a heavier anion was responsible for a high first-order static hyperpolarizability (β) and second-order susceptibility [χ^(2)^]. We also analysed similar MOFs with similar composition (fructose, Sr and I) but different structures and stoichiometries (Marabello *et al.*, 2015 ▸, 2019*b* ▸). In all these cases, we observed that different arrangements of the same building blocks in the crystal structure play a fundamental role in determining the SHG efficiency, and, furthermore, some peculiar combinations of symmetry elements can cancel the SHG response even in an acentric structure.

The aim of the present work is to analyse the role of the sugar in determining the SHG properties in this type of MOF. In the past, by screening experimentally the SHG efficiencies of about 150 powdered saccharides, Bourhill *et al.* (1993 ▸) observed that a higher SHG signal was produced by those saccharides that crystallize in space groups with lower symmetry. Among them, 2-de­oxy-d-galactose (DGal) showed the most promising SHG response. Thus, we oriented our synthesis towards MOFs containing this saccharide, along with alkali earth halogenides, hoping that it would impart high SHG efficiency to the crystals.

DGal is a de­oxy hexose sugar known to interfere with the glycoprotein metabolism in the influenza virus (Klenk *et al.*,1972 ▸) and rat liver (Keppler *et al.*, 1970 ▸). It crystallizes in the polar space group *P*2_1_, with all the molecular units adopting the β-2-de­oxy-d-galactopyran­ose ring form (Puliti *et al.*, 1984 ▸) (see scheme). 
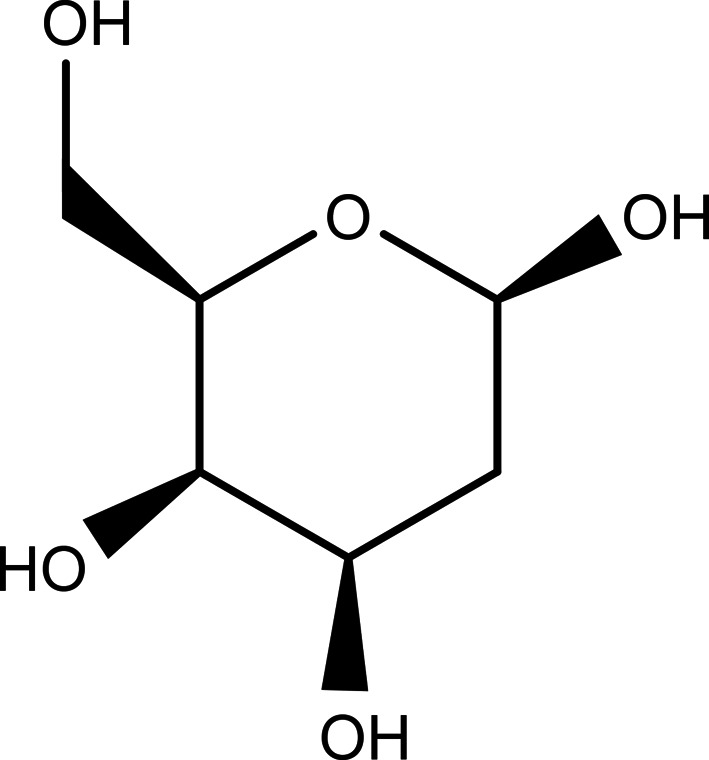


By combining 2-de­oxy-d-galactose with the halogenides Ca*X*_2_ (*X* = Br, I), we obtained two new isomorphous MOFs of formula [Ca(C_6_H_12_O_5_)_2_]*X*_2_, named CaDGalBr and CaDGalI, respectively, for which the SHG is expected to be higher than that of fructose-based MOFs, if the contribution of the sugar is what causes the SHG efficiency. In order to understand this perspective, the two compounds were characterized by single-crystal X-ray diffraction. The first-order static hyperpolarizability and second-order susceptibility were estimated by *in vacuo* and in-crystal density functional theory (DFT) calculations, and compared with experimental SHG values obtained for powdered samples.

## 2. Experimental   

### 2.1. Synthesis of [Ca(C_6_H_12_O_5_)_2_]Br_2_ (CaDGalBr)   

Calcium bromide and 2-de­oxy-d-galactose in stoichiometric ratios of 2:1 (0.200:0.075 g), 1:1 (0.100:0.075 g) and 1:2 (0.050:0.075 g) were dissolved in ethanol (1 g). The solvents were evaporated slowly at room temperature and after a few days colourless crystalline powders were formed. The powders were washed with a few drops of ethanol and dried in an oven at 323 K.

### 2.2. Synthesis of [Ca(C_6_H_12_O_5_)_2_]I_2_ (CaDGalI)   

Calcium iodide and 2-de­oxy-d-galactose in stoichiometric ratios of 2:1 (0.180:0.050 g), 1:1 (0.090:0.050 g) and 1:2 (0.045:0.050 g) were dissolved in ethanol (1 ml). The solutions were heated up to 353 K for 30 min, cooled to room temperature and the solvent was evaporated for two days in an oven at 323 K to give colourless crystalline powders. The powders were washed with a few drops of ethanol and dried in an oven at 323 K.

### 2.3. Single-crystal X-ray diffraction (XRD)   

X-ray diffraction data for CaDGalBr and CaDGalI were collected at room temperature using an Oxford Diffraction Gemini R Ultra diffractometer. Data were collected with mirror-monochromated Cu *K*α radiation (1.5418 Å). The *CrysAlisPro* (Agilent, 2014 ▸) package was used for data collection and integration, *SHELXT* (Sheldrick, 2015*a* ▸) for resolution, *SHELXL* (Sheldrick, 2015*b* ▸) for refinement and *OLEX2* (Dolomanov *et al.*, 2009 ▸) for graphics.

Crystal data for CaDGalBr (*M*_w_ = 564.24): monoclinic, space group *P*2_1_, *Z* = 2. Cell parameters are reported in Table 1 ▸. Reflections collected 5021, of which 2698 unique (*R*_int_ = 0.0374). *R*_1_ = 0.0350, *wR*_2_ = 0.0764 (all data).

Crystal data for CaDGalI (*M*_w_ = 658.22): monoclinic, space group *P*2_1_, *Z* = 2. Cell parameters are reported in Table 1 ▸. Reflections collected 11 076, of which 3737 unique (*R*_int_ = 0.0434). *R*_1_ = 0.0316, *wR*_2_ = 0.0792 (all data).

All atoms except H atoms were refined with anisotropic displacement parameters. Due to the low number of reflections collected, even if the H-atom peaks were observed in the difference Fourier maps, the H atoms were calculated and refined as riding with *U*_iso_ = 1.2 or 1.5 times *U*_eq_ of the connected carbon or oxygen atom. The interested reader can find further details of crystal data, data collection, least-squares refinements and bond lengths in the supporting information (Tables S1 and S2) and CIF files (CCDC 2058083 and 2058084).

### 2.4. Computational methods   

#### 2.4.1. *In vacuo* calculations   

The calculations were performed with the *GAUSSIAN09* and *GAUSSIAN16* set of programs (Frisch *et al.*, 2009 ▸, 2016 ▸). All the structures in this work were optimized by gradient-based techniques (Schlegel & Daudel, 1981 ▸; Schlegel, 1982*a* ▸,*b* ▸; Schlegel *et al.*, 1984 ▸) with no symmetry constraints at the density functional theory (DFT) B3LYP level of theory (Becke, 1988 ▸, 1993 ▸), in conjunction with the 6-31G(d) basis set, for the C, H, O, Ca and Br atoms (Hehre *et al.*, 1986 ▸). For iodine the LANL2DZ basis was used (Wadt & Hay, 1985 ▸). All critical points were characterized as energy minima by calculating their analytical frequencies. The total dipole moment, polarizability and first-order hyperpolarizability were calculated at the same level of theory. Molecular volumes were computed by averaging ten different volume calculations on the optimized geometries at the B3LYP level of theory with the options scf = tight, volume = tight and iop(6/45 = 500,6/46 = 1) (Parsons & Ninham, 2009 ▸).

#### 2.4.2. Bulk calculations   

DFT quantum simulations in the solid state were performed with the linear combination of Gaussian-type function (LCGTF) approach as implemented in the *CRYSTAL14* package (Dovesi *et al.*, 2014*a* ▸). According to the results of our former study (Marabello *et al.*, 2019*b* ▸), the hybrid PBE0 hamiltonian (Adamo & Barone, 1999 ▸) was selected throughout. C, H and O atoms were modelled with a 6-31G* split-valence basis set optimized for molecular crystals (Spackman & Mitchell, 2001 ▸). A Doll–Stoll large-core pseudopotential was applied to the bromide and iodide species (Doll & Stoll, 1998 ▸), while Ca^2+^ ions were described by a Kaupp small-core pseudopotential (Kaupp *et al.*, 1991 ▸; Kulkarni *et al.*, 2010 ▸). All the simulations relied on X-ray derived structures, with atomic coordinates having been fully relaxed at fixed experimental unit-cell parameters. Coupled perturbed (CP) Kohn–Sham calculations were then used to estimate the first- and second-order polarizabilities (Ferrero *et al.*, 2008*a* ▸,*b* ▸,*c* ▸). The same quantum simulations were also carried out on crystalline sucrose (Gražulis *et al.*, 2009 ▸; Russo *et al.*, 2013 ▸) and 2-de­oxy-β-galactose (Puliti *et al.*, 1984 ▸), taken as references for estimating the relative SHG response of CaDGalBr and CaDGalI. The interested reader can find full technical details of the computational procedure in Section S2 of the supporting information.

### 2.5. SHG measurements   

The SHG efficiency of the powdered compounds was measured by the method of Kurtz & Perry (1968 ▸). Samples were ground in an agate mortar (grain sizes below 100 µm) and heated in an oven at 323 K to avoid the absorption of humidity, before being sealed into capillaries.

The non-resonant 1064 nm wavelength of a Nd:YAG pulsed laser beam was directed onto capillaries containing the samples. The scattered radiation was collected by an elliptical mirror, filtered to select only the second-order contribution at 532 nm (*I* ^2ω^), and re-collected with a Hamamatsu R 5108 photomultiplier tube. The SHG efficiency was evaluated by taking as reference the SHG signal of ground sucrose powders (*I* ^2ω^/*I* ^2ω^_sucrose_).

## 3. Results and discussion   

### 3.1. Synthesis and crystal structures   

Both compounds were synthesized through a simple procedure, as were the analogous compounds of our previous studies, by dissolution of the reagents in ethanol and subsequent evaporation of the solvent. For each compound, three solutions with salt:sugar stoichiometric ratios of 2:1, 1:1 and 1:2 were prepared and after few days a white crystalline precipitate was observed. The only difference between the syntheses of the two compounds was that in the case of CaDGalI the solutions were heated up to 353 K for 30 min and maintained at 323 K during evaporation, to avoid the formation of the I_3_^−^ ion. The dried powders are slightly hygroscopic in humid air and stable below 353 K; above this temperature they degrade to a dark-brown amorphous powder.

Crystals suitable for X-ray structure determination were obtained for both compounds from the solutions with a stoichiometric ratio of 1:1. To avoid the absorption of water by the mounted crystals they were covered with a paraffin oil for the X-ray measurements.

The two compounds are almost perfectly isostructural (root-mean-square deviation or r.m.s.d. = 0.163 Å) and do not exhibit any disorder. As expected, the I-containing crystal has a slightly larger (by 6.9%) cell volume, but the large iodine ions do not imply any significant change in the packing motifs (Table 1 ▸). Thus, any difference in the optical behaviour is expected to be due to the different chemical nature of the polarizable halogen atoms.

**Figure 1:**
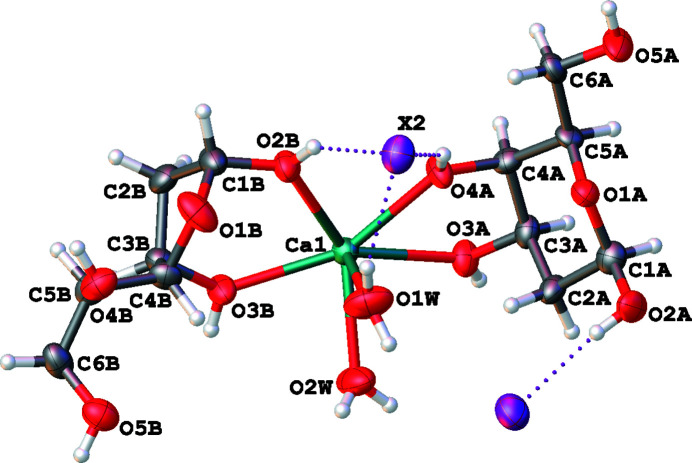
The asymmetric unit of both compounds (*X* = Br, I). Displacement ellipsoids are drawn at the 50% probability level and H atoms are shown as small spheres of arbitrary radii. Dotted lines indicate O⋯*X* hydrogen bonds.

**Figure 2:**
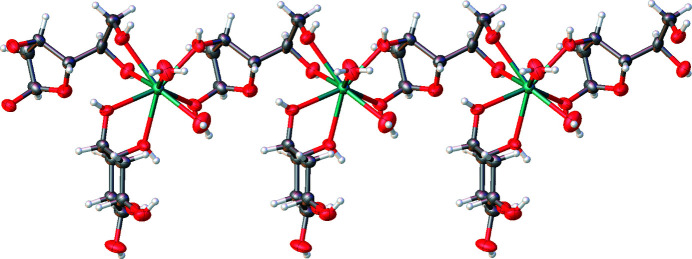
Chains of metals and sugar molecules developed in the [100] direction.

The asymmetric unit of both compounds consists of one Ca^2+^ ion, two *X* ^−^ anions, two sugar molecules and two water molecules coordinated to the metal atom (Fig. 1 ▸). One sugar molecule adopts the α-d-pyran­ose form, the other the β-d-furan­ose form. It is worth noting that at equilibrium in aqueous solution the two cyclic forms coexist, with a pre­dominance of the six-membered one (∼5:1) (Angyal & Pickles, 1972 ▸). Our conditions clearly shift the equilibrium toward a 1:1 ratio of pyran­ose:furan­ose forms for both compounds. This is likely to be due to a metal-mediated template effect, as β-2-de­oxy-d-galactofuran­ose is a more effective chelating agent. Each furan­ose ring can bind two Ca^2+^ ions by exploiting at the same time the exocyclic hydroxyl groups on the anomeric side, and the aliphatic ones on the opposite side, forming extended Ca–furan­ose chains that run along the *a* axis (Fig. 2 ▸). Thus, the structures are classified as 1D-MOFs. Each Ca^2+^ ion is also chelated by a pyran­ose ring that does not bridge to any other cation. Pyran­ose rings are too large to fill the space around the cations effectively, and in fact they are arranged orthogonally with respect to the metal–organic chains, along the *b* direction (Fig. 2 ▸, and Fig. S7 in the supporting information). In the end, the coexistence of smaller and larger rings in this structure allows an efficient occupation of space, in agreement with Kitaigorodskii’s principle of close packing (Kitaigorodskii, 1961 ▸).

One-dimensional chains were also observed in some of analogue MOF structures containing fructose and alkali earth halogenides (Marabello *et al.*, 2017 ▸, 2019*b* ▸). In these cases, two metal cations were bridged by two fructose mol­ecules, while in the present compounds and in [Sr(fructose)(H_2_O)_3_I]I (Marabello *et al.*, 2019*b* ▸) only one sugar molecule bridges two metal cations.

**Figure 3:**
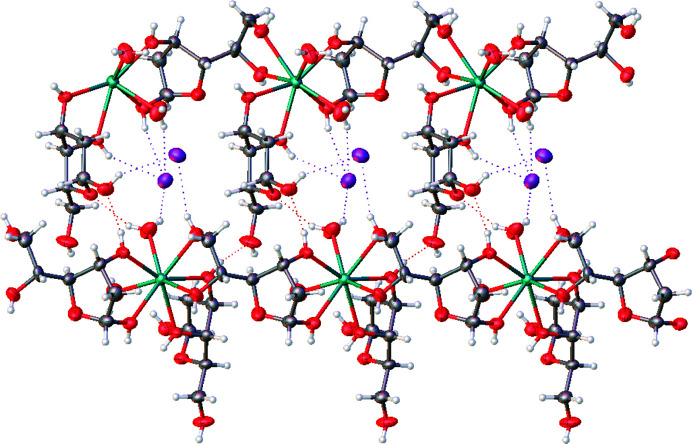
Two adjacent chains connected through several hydrogen bonds, shown as dotted lines. Atom labelling is as reported in Fig. S1 of the supporting information).

In the present compounds, the Ca–furan­ose parallel chains are reciprocally connected in the crystal through strong hydrogen bonds involving the halogen anion, the water molecules and the free OH groups of the pyran­ose sugar molecules (Fig. 3 ▸, and Table S3 and Fig. S1).

### 3.2. Computational results   

For experimental applications, the MOFs have to be reduced to particles of a few tens of nanometres, which can be obtained by vigorously grinding the crystals. In fact, in our previous work (Marabello *et al.*, 2019*a* ▸) on analogous structures (two MOFs composed of Sr^2+^, fructose and Cl^−^/I^−^) we demonstrated the inverse proportionality between the size of the ground particles and the grinding energy of a planetary mill (grinding time and number of revolutions per minute).

Therefore, it is important to ascertain the SHG behaviour of small fragments of the compounds analysed, by considering the structural distortion that the surface forces can induce at the nanoscale level.

#### 3.2.1. *In vacuo* computational results   

**Figure 4:**
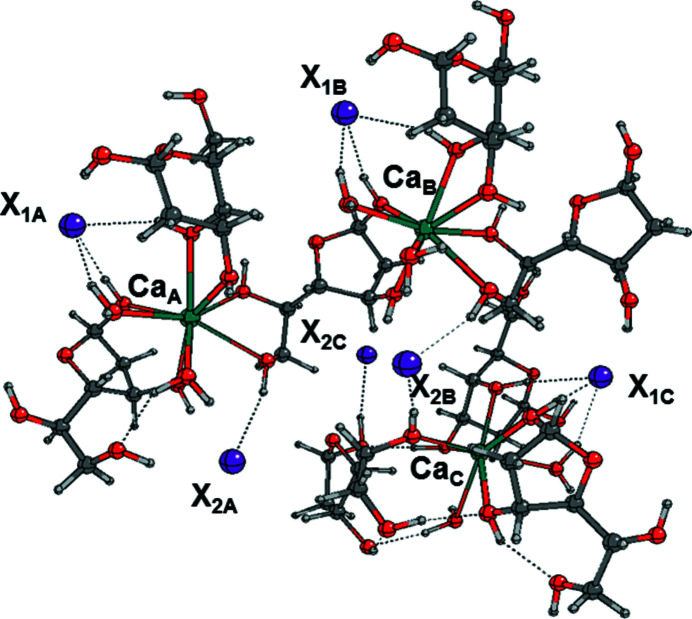
Fragment1 of the crystal structure of CaDGgal*X* (*X* = Br,I), optimized at the B3LYP level of theory. Dotted lines indicate hydrogen bonds.

To this end, two different small fragments of the crystal were selected (Figs. 4 ▸ and 5 ▸) and the relevant geometries were recomputed by optimizing the atomic coordinates derived from the X-ray structures.

**Figure 5:**
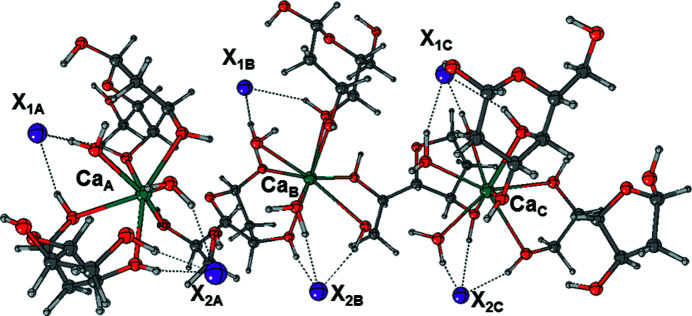
Fragment2 of the crystal structure of CaDGgal*X* (*X* = Br, I), optimized at the B3LYP level of theory. Dotted lines indicate hydrogen bonds.

The composition of the fragments does not reflect their stoichiometry, but an excess of sugar molecules was added at the boundary of the structure to attain full coordination of the metal. Fragment1 in Fig. 4 ▸ is selected by cutting the crystal along the 1D Ca–furan­ose chain and is composed of three calcium ions, eight 2-d-galactose molecules, six anions (Br^−^ or I^−^) and six water molecules, while Fragment2 in Fig. 5 ▸ is obtained by cutting the crystal along two parallel Ca–furan­ose chains, connected through several hydrogen bonds, and is composed of three calcium ions, seven 2-d-galactose mol­ecules, six anions (Br^−^ or I^−^) and six water molecules. Since sucrose is the usual reference compound for SHG measurements, the same types of calculation were carried out on a model of bulk sucrose, composed of four sucrose units. The atomic coordinates of sucrose were obtained from the Cambridge Structural Database (CSD; Russo *et al.*, 2013 ▸). All geometries were re-optimized at the B3LYP level of theory to obtain the corresponding minima.

Tables 2 ▸ and 3 ▸ show the most relevant geometric parameters compared with the corresponding X-ray data for the two fragments.

As expected, the optimized structural parameters show deviations from the corresponding X-ray data, probably due to the small size of the computed fragments, which involve a certain degree of asymmetry with respect to the crystal. The most relevant differences between the X-ray data and the theoretical calculations are found in the bonds between Ca^2+^ and the coordinated O atoms and range from 0.01 Å to a maximum of 0.59 Å in the CaDGalBr Fragment1. In Fragment2, the bond differences are smaller and range from 0.07 Å to a maximum of 0.20 Å. The same trend is observed in the CaDGalI complex, where the differences range from 0.04 to 0.69 Å in Fragment1 and decrease in Fragment2, ranging from 0.05 to 0.21 Å. Greater differences are observed in the distances between the Ca^2+^ ions and between Ca cations and *X* anions: the differences in the Ca^2+^⋯Ca^2+^ distances range from a minimum of 1.0 Å to 2.4 Å and in the Ca^2+^⋯*X* ^−^ distances from 0.6 to 1.1 Å. The differences between the experimental and theoretical calculation results are probably caused by the difficulty that DFT with double-ζ basis sets has to describe the non-covalently bound entities.

Table S4 collects the results of natural bond orbital (NBO) calculations: the natural atomic charges on Ca, Br and I and the group charges of the galactose and water molecules are reported. No significant differences were observed between the complexes or between the different fragments.

Table 4 ▸ shows the computed values of the dipole moment μ, the mean polarizability α, the first static hyperpolarizability β_tot_ and the second-order susceptibility χ^(2)^ for the two compounds and the two different fragments. The ratio between the second-order susceptibility of the compounds to that of sucrose is also reported, in order to compare the computational results with the experimental second harmonic measurements.

The total dipole moments μ and the mean polarizabilities α in a Cartesian frame are defined as:

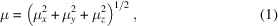




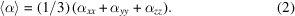

The total intrinsic hyperpolarizability β_tot_ is defined as:

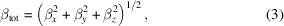

where 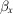 = 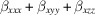 , 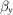 = 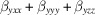 and 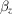 = 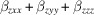 .

The relationship between the macroscopic second-order susceptibility, the quantity that correlates to the second-harmonic intensity, and the microscopic total hyperpolarizability is given by equation (4),

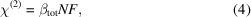

where *N* is the number of particles per unit volume and *F* is the local field factor. *F* depends upon the crystal symmetry. It is related to the crystal’s refractive index, and it can vary if different compounds are considered. Values between 1 and 2 are generally reported (Choudhury & Chitra, 2011 ▸) and in particular for saccharides this value is close to 1.0. Furthermore, since the compounds studied have the same structure, we expect their refractive indices to be equal. Thus, since our interest is focused on the trend of χ^(2)^ values, we assumed *F* = 1.

In Table 4 ▸, we observe that for both fragments the values of the total intrinsic hyperpolarizability are very different for the two complexes, *i.e.* the value of β_tot_ for the iodine complex is twice the value of the bromine complex. For Fragment1 this trend correlates with the substantial decrease in the frontier orbitals gap of the iodine complex. The same trend is not observed for Fragment2.

The calculated static susceptibility χ^(2)^ values are similar for the two fragments of the same complex, underlying that the geometry of the fragments does not affect this result, while a difference is observed by comparing the χ^(2)^ values of the Br^−^ versus I^−^ complexes. The complexes containing the larger and more polarizable I^−^ anions show a higher value of χ^(2)^, confirming the trend already observed in our previous work.

#### 3.2.2. Bulk computational results   

Bulk coupled perturbed Kohn–Sham (CPKS) DFT simulations (Section 2.4, and Section S2.1 in the supporting information) included the coupling of an external electric field with the crystal field, allowing us to extract from the Bloch-consistent periodic wavefunction information on optical axes, dielectric tensors and first- and second-order polarizabilities. This approach bears several advantages against the more classical sum-over-state (SOS) method under Unsöld’s approximations (Unsöld, 1927 ▸). One of these advantages is that CPKS simulations allow the wavefunction to relax self-consistently under a perturbing electric field. Moreover, the Born–von Karman boundary conditions account for the periodicity of the crystal structure. The DFT-optimized crystal structures are fully consistent with the experimental X-ray ones (Figs. S4–S5): the r.m.s.d.s on the coordinates of C, O, Ca^2+^ and halogen atoms within the whole unit cell do not exceed 0.12/0.28 Å in the compounds CaDGalBr/CaDGalI. As expected, the largest deviations affect H atoms, and particularly the relative orientation of Ca-coordinated water molecules [Figs. S4(*a*)–S4(*b*) and S5(*a*)–S5(*b*)]. However, the main structural and coordination features discussed above are fully preserved.

Since CaDGalBr and CaDGalI are almost perfectly isostructural, any difference in their optical behaviour is expected to be due to the different chemical nature of the polarizable halogen atoms. Indeed, NLO properties in these structures cannot be rationalized in terms of simple geometric/charge-transfer models, like in push–pull systems (Beverina *et al.*, 2011 ▸). The nonlinear response is mostly due to the large polarizability of the halogen (Marabello *et al.*, 2017 ▸). Therefore, no significant bond-length alternation (BLA) effects are detectable in the sugar. Moreover, all the NLO measurements were carried out on micrometre-to-millimetre sized grains. Thus, the average crystallite dimensions are larger than the coherence length, making the second-harmonic efficiencies independent, on average, of the particle size (Marabello *et al.*, 2019*b*; Bourhill *et al.*, 1993 ▸). Under these conditions, the second-order intensity is proportional to the square averaged 〈(*ijk*)^2^〉 second-order polarizability tensor elements, which can be estimated from DFT bulk calculations.

Table 5 ▸ displays the predicted first- and second-order responses of CaDGalBr and CaDGalI, in comparison with sucrose (Bourhill *et al.*, 1993 ▸) and DGal sugars. According to the procedure developed by Marabello *et al.* (2019*b* ▸), a weighted average of second-order matrix elements was carried out based on *P*2_1_-compatible symmetry multiplicities (three for *xxy* and *yyz*, six for *xyz* and one for *yyy*). All averages were computed from the squared *d*_*ijk*_ elements in MKS units, and the corresponding ratios with respect to sucrose, 〈*d*_*ijk*_^2^〉_sucrose_, were evaluated (Table 6 ▸).

### 3.3. SHG results and comparison with theoretical calculations   

The SHG values of the two compounds obtained from Kurtz–Perry measurements on ground powders of sizes below 100 µm and from theoretical calculations performed both *in vacuo* and in bulk are reported in Table 6 ▸. A qualitative agreement is observed among the B3LYP and bulk DFT predictions and the experimental outcomes.

The experimental SHG efficiency of DGal previously reported by Bourhill *et al.* (1993 ▸) equals 3.9 times that of sucrose. The orders of magnitude of the experimental values obtained using our modified Kurtz–Perry setup agree with those of Bourhill *et al.* (1993 ▸), except that the measured efficiency of DGal is only slightly higher than that of sucrose.

By comparing the results in Table 6 ▸ it turns out that, for both the calculations (*in vacuo* and in bulk) and the experimental measurements, the SHG response of the two compounds is approximately the same as DGal itself. For the bulk calculations, it is noticeable that there is a slightly higher difference between the values of the two MOFs with respect to the experimental measurements. This, however, is to be expected, considering that experiments are carried out in conditions of non-ideality, while DFT calculations refer instead to static (no thermal motion) geometries at 0 K in the limit of static (no time-dependency) high-frequency (only electronic contributions) dielectric susceptibilities. Moreover, the experiments were carried out on powdered samples, to be confronted with the infinite perfect lattices of our CPKS model. Therefore, bulk simulations lack thermal motion, dispersive behaviour of refractive indices at finite wavelengths and possible iso-orientations of crystallites, and the observed discrepancies are probably imputable to the intrinsic limits of the computational approaches. The take-home message from Table 6 ▸ is that there is a general qualitative agreement between experiment and theory, as the predicted and observed susceptibilities are roughly of the same order of magnitude.

A closer comparison of the theoretical results alone (first two rows of Table 6 ▸) shows that the three systems follow the same trend, irrespective of the length scale, as both molecular clusters and bulk simulations rank the second-order susceptibilities in the following order: CaDGalBr < DGal < CaDGalI. However, in bulk systems the expected response is from ∼2 to ∼4 times higher. There is a 1:1 correspondence among the X-ray observed crystal structures and the model we employed to perform bulk calculations (see Section 3.2 above). Therefore, the enhancement of the predicted NLO response of the bulk calculations with respect to the isolated clusters is entirely ascribable to crystal field effects.

Different from our previous findings for fructose-containing derivatives, the involvement of DGal in a MOF structure has no significant influence on the NLO response. However, in this case we are in a different crystallographic situation: fructose crystallizes in a more symmetric structure with respect to its calcium MOFs (orthorhombic versus monoclinic) while the DGal sugar crystallizes in the same monoclinic space group (*P*2_1_) as CaDGal*X* MOFs. This observation confirms the assertion that the SHG response is principally influenced by the symmetries in the structure: the lower the symmetries the higher the SHG efficiency. As the three materials share the same crystal symmetry and have similar packing features, it should be expected that they produce similar NLO outcomes. From a structural viewpoint, the average ratios (〈*B*/*A*〉) of imaginary and real contributions to the structure-factor amplitudes of DFT-predicted nonextinct reflections within sinθ/λ = 0.55 Å^−1^ are identical [DGal 3.7 (8), CaDGalBr 3.8 (5) and CaDGalI 3.7 (5)]. Thus, symmetry breaking is not the main trigger of the NLO response. This is consistent with our Kurtz–Perry measurements, even though the bulk DFT simulations predict that, under ideal conditions, the iodine chromophore should perform better than the bromine one (Table 6 ▸). Any difference in the performance of these isomorphous crystal architectures should be ascribed to electronic reasons. However, further studies are needed to shed light on how electronic and chemical degrees of freedom are related to the crystal symmetry.

As mentioned above, we expected that substituting fructose with DGal would impart a higher SHG efficiency to the *M*(sugar)*X* MOFs. Instead, the two DGal MOFs analysed in this work do not show the expected enhancement of SHG efficiency with respect to the analogous fructose-based ones studied in our previous work. Thus, the nature of the sugar can influence the SHG behaviour of the compounds only because it entails a change in the crystal structure.

The SHG measurements and the theoretical calculations agree on the fact that the SHG efficiency of CaDGalI is greater than that of CaDGalBr. Since the two compounds are isostructural and isomorphous, this behaviour is necessarily imputable to the larger polarizability of iodine ions and was observed in all the isostructural and isomorphous compounds previously analysed. It is noteworthy that in the bulk calculations the difference in the responses of the two MOFs is greater than for the other two methods. This behaviour might be traced back to cooperativity effects, that is, to the symmetry-constrained alignment of polar molecules in the bulk crystal. It is known, for example, that an external electric field can partially align polar mol­ecules, eliciting a temperature-dependent contribution to SHG even in an otherwise isotropic achiral liquid (Wagnière & Woźniak, 2017 ▸).

## 4. Conclusions   

In this work we analysed the SHG efficiency of two iso­structural and isomorphous DGal-derived MOFs with respect to the sugar itself and similar fructose-derived MOFs analysed previously. Based on the results, we can conclude that the nature of the sugar present in this kind of MOF does not significantly affect the SHG response: the most important role of the sugar is to cause the absence of an inversion centre and to determine a change in the structural arrangements. Instead, the lower symmetry in the structure seems to have a fundamental role in the SHG efficiency, and likewise the presence of the more polarizable iodide ion. Furthermore, the bulk calculations suggest that, in principle, large bulk crystals should show a higher SHG response than isolated molecular clusters or nanoparticles.

In conclusion, even though the compounds analysed show an SHG efficiency comparable with that of sucrose and can thus be usefully applied as bio-sensors, the results of this work suggest that we can try to improve the SHG efficiency by suitably modulating the symmetry of the crystal structure and the chemical composition, *i.e.* less symmetric structures with more polarizable anions.

## 5. Related literature   

For further literature related to the supporting information, see Dovesi *et al.* (2014*b* ▸), Broyden (1965 ▸), Johnson (1988 ▸), Lacivita *et al.* (2016 ▸, 2012 ▸), Cremer & Pople (1975 ▸) and Boeyens (1978 ▸).

## Supplementary Material

Crystal structure: contains datablock(s) CaDgalBr_new, CaDgalI_new. DOI: 10.1107/S2052520621004637/um5049sup1.cif

Structure factors: contains datablock(s) CaDgalBr_new. DOI: 10.1107/S2052520621004637/um5049CaDgalBrsup2.hkl

Structure factors: contains datablock(s) CaDgalI_new. DOI: 10.1107/S2052520621004637/um5049CaDgalIsup3.hkl

Additional details, including refinements, calculations and hydrogen bonds. DOI: 10.1107/S2052520621004637/um5049sup4.pdf

CCDC references: 2058083, 2058084

## Figures and Tables

**Table 1:** Cell parameters for the DGal ligand and the compounds CaDGalBr and CaDGalI

	DGal†	CaDGalBr	CaDGalI
Crystal system	Monoclinic	Monoclinic	Monoclinic
Space group	*P*2_1_	*P*2_1_	*P*2_1_
*a* (Å)	9.811 (1)	7.5022 (4)	7.6384 (2)
*b* (Å)	6.953 (1)	14.2259 (6)	14.4621 (4)
*c* (Å)	5.315 (1)	10.4520 (6)	10.7490 (3)
β (°)	91.58 (2)	109.931 (6)	109.233 (4)
Volume (Å^3^)	362.43 (9)	1048.7 (1)	1121.14 (6)

† Literature data from CSD refcode DACHIY (Puliti *et al.*, 1984 ▸).

**Table 2:** Relevant distances (Å) around the metal atom in Fragment1 from X-ray data and B3LYP/6-31G(d) calculations

	CaDGalBr		CaDGalI	
	XRD	B3LYP	XRD	B3LYP
Ca1—O1*W*	2.444 (7)	2.458	2.447 (8)	3.152
Ca1—O2*B*	2.491 (6)	2.520	2.478 (6)	2.534
Ca1—O2*W*	2.409 (7)	2.550	2.388 (7)	2.507
Ca1—O3*A*	2.471 (6)	2.586	2.468 (6)	2.556
Ca1—O3*B*	2.391 (6)	2.528	2.429 (6)	2.617
Ca1—O4*A*	2.466 (6)	2.548	2.471 (5)	2.541
Ca1—O4*B*1	2.414 (6)	2.474	2.415 (6)	2.460
Ca1—O5*B*1	2.504 (6)	3.100	2.500 (7)	2.554
Ca^2+^⋯Ca^2+^ (average)	9.665	8.048	11.162	8.767
Ca^2+^⋯*X* ^−^ (average)	5.207	4.568	5.805	5.133

**Table 3:** Relevant distances (Å) around the metal atom in Fragment2 from X-ray data and B3LYP/6-31G(d) calculations

	CaDGalBr		CaDGalI	
	XRD	B3LYP	XRD	B3LYP
Ca1—O1*W*	2.444 (7)	2.511	2.447 (8)	2.516
Ca1—O2*B*	2.491 (6)	2.627	2.478 (6)	2.618
Ca1—O2*W*	2.409 (7)	2.487	2.388 (7)	2.499
Ca1—O3*A*	2.471 (6)	2.601	2.468 (6)	2.680
Ca1—O3*B*	2.391 (6)	2.486	2.429 (6)	2.472
Ca1—O4*A*	2.466 (6)	2.594	2.471 (5)	2.596
Ca1—O4*B*1	2.414 (6)	2.493	2.415 (6)	2.497
Ca1—O5*B*1	2.504 (6)	2.710	2.500 (7)	2.687
Ca^2+^⋯Ca^2+^ (average)	7.503	6.492	7.638	6.417
Ca^2+^⋯*X* ^−^ (average)	4.968	4.370	5.508	4.363

**Table 4:** *In vacuo* computed dipole moments μ (Debye), mean polarizabilities 〈α〉 (a.u.), first-order static hyperpolarizabilities β_tot_ (10^−30^ cm^5^ esu^−1^), second-order susceptibilities χ^(2)^ (pm V^−1^), second-order susceptibility ratios, *E*_HOMO,_ *E*_LUMO_ and Δ*E* (a.u.) with respect to sucrose values

	Fragment1		Fragment2		
	CaDGalBr	CaDGalI	CaDGalBr	CaDGalI	DGal
μ	24.93	26.43	21.57	19.41	26.13
〈α〉	868.442	880.400	782.787	807.653	681.773
β_tot_	9.4	14.9	7.8	14.3	7.4
χ^(2)^	1.55	2.28	1.42	2.35	1.55
χ^(2)^/χ^(2)^_sucrose_	0.85	1.25	0.78	1.29	0.85
*E*_HOMO_	−0.20328	−0.19556	−0.22553	−0.20715	−0.19248
*E*_LUMO_	−0.02593	−0.03832	−0.03929	−0.02404	−0.15356
Δ*E*	0.17735	0.15724	0.18624	0.18311	0.03892

**Table 5:** DFT-derived first-order electric susceptibilities [χ^(1)^, dimensionless], diagonalized dielectric tensor elements (ɛ, dimensionless†) and second-order electric susceptibilities [χ^(2)^, atomic units‡] for bulk CaDGalBr, CaDGalI, sucrose and 2-de­oxy-β-D-galactose (DGal), all in space group *P*2_1_

	CaDGalBr	CaDGalI	Sucrose§	DGal
χ_*xx*_^(1)^	1.0553	1.0263	1.1622	1.1580
χ_*xz*_^(1)^	0.01	0.0306	−0.0435	−0.0563
χ_*yy*_^(1)^	1.0901	1.1064	1.2136	1.2302
χ_*zz*_^(1)^	1.0078	1.0893	1.1750	1.1576
ɛ_11_	2.0573	2.0138	2.1247	2.2141
ɛ_22_	2.0901	2.1064	2.2136	2.2302
ɛ_33_	2.0057	2.1017	2.2125	2.1015
χ_*xxy*_^(2)^	−0.0664	0.2583	0.1447	−0.1493
χ_*xyz*_^(2)^	0.1552	0.1050	−0.0013	−0.1954
χ_*yyy*_^(2)^	0.2424	0.3996	0.2885	−0.4466
χ_*yzz*_^(2)^	0.2434	0.6075	0.2048	−0.0472

† Diagonal elements of the dielectric tensor, ɛ, in the principal axes system.

‡ Second-order susceptibilities can be expressed in other conventions through the usual conversion factors. Frequent alternative expressions of the second-order tensor components as β_*ijk*_ or *d_ijk_* quantities (always in atomic units) are β_*ijk*_ = (*V*χ_*ijk*_)/2π, *V* being the unit-cell volume in cubic bohr, and *d_ijk_* = χ_*ijk*_/2. Conversion to the MKS system in terms of reciprocal electric field units can be accomplished according to *d_ijk_* (MKS) = *d_ijk_* (a.u.)/0.514220632 pm V^−1^. See also https://physics.nist.gov/cuu/Constants/index.html.

§ DFT estimates for sucrose at the same level of theory employed in this work have been taken from Marabello *et al.* (2019*b* ▸).

**Table 6:** Ratio between the average second-order susceptibilities of the two fragments with respect to the sucrose values obtained from *in vacuo* calculations, ratio between the average second-order squared susceptibility tensor elements with respect to crystalline sucrose as estimated through bulk calculations, and ratio between the second-harmonic signal at 532 nm produced by powdered samples and that of standard sucrose under the same experimental conditions, for compounds CaDGalBr, CaDGalI and 2-de­oxy-β-D-galactose

		CaDGalBr	CaDGalI	DGal
*In vacuo* (DFT:B3LYP)	χ^(2)^/χ^(2)^_sucrose_	0.82	1.27	0.85
Bulk (DFT:PBE0)	〈*d_ijk_* ^2^〉/〈*d_ijk_* ^2^〉_sucrose_	1.50	5.60	1.80
SHG measurements	*I* ^2ω^/*I* ^2ω^_sucrose_	0.64	0.78	1.20
