# Supplementary material for: Crystal structure or chemical composition of salt–sugar-based metal–organic frameworks: what are the nonlinear optical properties due to?
Source: Acta Crystallogr B Struct Sci Cryst Eng Mater. 2021 Jul 6;77(Pt 4):506–14. doi: 10.1107/S2052520621004637 (PMC8337021; doi:10.1107/S2052520621004637)
Supplement: Supplementary file 4 [file b-77-00506-sup4.pdf]

Acta Cryst B
STRUCTURAL SCIENCE
CRYSTAL ENGINEERING
MATERIALS

Volume 77 (2021)

Supporting information for article:

Crystal structure or chemical composition of salt–sugar-based metal–organic frameworks: what are the nonlinear optical properties due to?

Domenica Marabello, Paola Antoniotti, Paola Benzi, Fabio Beccari, Carlo Canepa, Elena Cariati, Alma Cioci and Leonardo Lo Presti

Section S1. Details of X-ray structures and refinements and B3LYP calculations

Table S1. Details on crystal data and structure refinement for CaDGalBr and CaDGalI

	**CaDGALBr**		**CaDGalI**
Empirical formula	$C_{12}H_{28}O_{12}CaBr_2$		$C_{12}H_{28}O_{12}CaI_2$
Formula weight	564.24		658.22
Temperature/K		293(2)	
Crystal system		monoclinic	
Space group		$P2_1$	
a/Å	7.5022(4)		7.6384(2)
b/Å	14.2259(6)		14.4621(4)
c/Å	10.4517(6)		10.7490(3)
β/°	109.931(6)		109.233(4)
Volume/Å^3	1048.65(10)		1121.14(6)
Z		2	
ρ_{calc}g/cm^3	1.787		1.950
μ/mm^{-1}	7.561		24.519
F(000)	572.0		644.0
Crystal size/mm^3	0.286 × 0.129 × 0.026		0.253 × 0.107 × 0.081
Radiation		CuKα (λ = 1.54184)	
2Θ range for data collection/°	9 to 117.868		8.712 to 133.828
Index ranges	$-8 \leq h \leq 7$<br>$-15 \leq k \leq 15$<br>$-10 \leq l \leq 11$		$-8 \leq h \leq 7$<br>$-17 \leq k \leq 17$<br>$-11 \leq l \leq 12$
Reflections collected	5021		11076
Independent reflections	2698		3737
R_{int}	0.0374		0.0434
R_{sigma}	0.0557		0.0439
Data/restraints/parameters	2698/17/254		3737/1/257
Goodness-of-fit on F^2	1.057		1.039
Final R_1index [I>=2σ (I)]	0.0350		0.0316
Final wR_2 index [I>=2σ (I)]	0.0713		0.0780
Final R_1index [all data]	0.0420		0.0332
Final wR_2 index [all data]	0.0764		0.0792
Largest diff. peak/hole / e Å^{-3}	0.33/-0.28		0.50/-0.80
Flack parameter	-0.017(18)		-0.016(9)

Table S2. Bond lengths for CaDGALBr and CaDGALI obtained from X-ray diffraction and from B3LYP calculations. All distances are averaged for the three equivalent atoms present in the fragments. Label (1) refers to Fragment1, label (2) to Fragment2.

		**CaDGalBr**			**CaDGalI**	
**Bond**	**X-ray**	**B3LYP(1)**	**B3LYP(2)**	**X-ray**	**B3LYP(1)**	**B3LYP(2)**
Ca1-O1W	2.444(7)	2.458	2.511	2.447(8)	3.152	2.516
Ca1-O2B	2.491(6)	2.520	2.627	2.478(6)	2.534	2.618
Ca1-O2W	2.409(7)	2.550	2.487	2.388(7)	2.507	2.499
Ca1-O3A	2.471(6)	2.586	2.601	2.468(6)	2.556	2.680
Ca1-O3B	2.391(6)	2.528	2.486	2.429(6)	2.617	2.472
Ca1-O4A	2.466(6)	2.548	2.594	2.471(5)	2.541	2.596
Ca1-O4B[1]	2.414(6)	2.474	2.493	2.415(6)	2.460	2.497
Ca1-O5B[1]	2.504(6)	3.100	2.710	2.500(7)	2.554	2.687
O1A-C1A	1.43(1)	1.429	1.433	1.43(1)	1.429	1.434
O1A-C5A	1.440(9)	1.426	1.426	1.43(1)	1.428	1.426
O1B-C1B	1.40(1)	1.414	1.428	1.39(1)	1.412	1.420
O1B-C4B	1.44(1)	1.446	1.443	1.45(1)	1.441	1.443
O2A-C1A	1.40(1)	1.386	1.384	1.39(1)	1.390	1.384
O2B-C1B	1.43(1)	1.433	1.422	1.43(1)	1.429	1.423
O3A-C3A	1.441(9)	1.449	1.453	1.429(9)	1.447	1.453
O3B-C3B	1.44(1)	1.445	1.431	1.42(1)	1.434	1.430
O4A-C4A	1.44(1)	1.443	1.443	1.43(1)	1.444	1.443
O4B-C5B	1.44(1)	1.428	1.427	1.44(1)	1.426	1.436
O5A-C6A	1.43(1)	1.437	1.413	1.43(1)	1.419	1.412
O5B-C6B	1.43(1)	1.437	1.440	1.42(1)	1.437	1.439
C1A-C2A	1.52(1)	1.540	1.546	1.52(1)	1.540	1.541
C1B-C2B	1.49(1)	1.528	1.524	1.50(1)	1.530	1.523
C2A-C3A	1.52(1)	1.528	1.529	1.51(1)	1.530	1.529
C2B-C3B	1.51(1)	1.529	1.536	1.51(1)	1.538	1.536
C3A-C4A	1.50(1)	1.532	1.529	1.52(1)	1.530	1.529
C3B-C4B	1.53(1)	1.547	1.558	1.54(1)	1.032	1.558
C4A C5A	1.54(1)	1.532	1.529	1.53(1)	1.533	1.529
C4B C5B	1.53(1)	1.528	1.530	1.52(1)	1.524	1.529
C5A-C6A	1.50(1)	1.526	1.528	1.51(1)	1.533	1.528
C5B-C6B	1.50(1)	1.526	1.529	1.50(1)	1.526	1.529

[1]-1+X,+Y,+Z

Table S3. Hydrogen bonds observed in CaDGalX from X-ray structures: X=Br, first row; X=I, second row.

D H A site_symmetry	HA	DA	DHA
O1W-H1WA···X2	2.58<br>2.90	3.398(7)<br>3.714(9)	163.4<br>159.8
O1W-H1WB···O5A[1]	2.56<br>2.47	3.229(10)<br>3.24(1)	136.2<br>150.5
O2A-H2A···X1	2.49<br>2.65	3.255(6)<br>3.463(6)	155.6<br>174.2
O2B-H2B···X2	2.54<br>2.65	3.283(6)<br>3.460(7)	151.6<br>168.9
O2W-H2WA···X2[1]	2.51<br>2.99	3.273(6)<br>3.450(7)	150.7<br>115.6
O2W-H2WB···O1A[1]	2.07<br>2.05	2.800(9)<br>2.829(9)	143.1<br>151.9
O3A-H3A···O5A[2]	2.08<br>2.09	2.818(8)<br>2.828(9)	148.8<br>148.9
O3B-H3B···O2A[1]	1.90<br>1.96	2.710(8)<br>2.764(8)	168.4<br>165.7
O4A-H4A···X2	2.56<br>2.84	3.319(6)<br>3.535(6)	149.0<br>143.9
O4B-H4B···X1[3]	2.60<br>2.80	3.410(6)<br>3.585(6)	168.8<br>160.1
O5A-H5A···X1[4]	2.46<br>2.68	3.272(7)<br>3.489(7)	170.5<br>169.1
O5B-H5B···X1[5]	2.59<br>2.78	3.368(6)<br>3.560(6)	158.7<br>158.6
C1B-H1B···O2A[3]	2.57<br>2.72	3.481(12)<br>3.64(1)	155.5<br>156.0
C4B-H4BA···O3A[6]	2.76<br>2.90	3.549(11)<br>3.68(1)	137.8<br>124.4

[1] -x, y-1/2, -z
[2] -x-1, y-1/2, -z
[3] -x, y+1/2, -z
[4] x+1, y, z+1

[5] -x+1, y-1/2, -z
[6] x+1, y, z

Table S4 : Results of NBO calculations: natural atomic charges on Ca, Br and I and group charges of galactose and water molecules for the two fragments (labels of groups are referred to Figures S2 and S3).

	**CaDGalBr**	**CaDGalI**		**CaDGalBr**	**CaDGalI**
**Fragment1** (*non* linear)			**Fragment2** (linear)		
Ca A	1.499	1.547	Ca A	1.503	1.540
X 1A	-0.712	-0.750	X 1A	-0.710	-0.743
X 2A	-0.747	-0.791	X 2A	-0.694	-0.733
Gal 1A	-0.008	-0.005	Gal 1A	-0.043	-0.017
Gal 2A	-0.047	-0.034	Gal 2A	-0.073	-0.042
H2O 1A	-0.03	-0.028	H2O 1A	-0.024	-0.024
H2O 2A	-0.011	-0.008	H2O 2A	-0.022	-0.018
Gal AB	0.030	0.012	Gal AB	-0.055	-0.011
Ca B	1.491	1.537	Ca B	1.519	1.557
X 1B	-0.710	-0.732	X 1B	-0.698	-0.723
X 2B	-0.708	-0.743	X 2B	-0.652	-0.706
Gal 1B	0.000	0.056	Gal 1B	0.011	-0.012
Gal 2B	0.012	-0.004	H2O 1B	-0.043	-0.034
H2O 1B	-0.036	-0.036	H2O 2B	-0.042	-0.034
H2O 2B	0.003	-0.010	Gal BC	-0.066	-0.070
Ca C	1.541	1.582	Ca C	1.502	1.526
X 1C	-0.803	-0.848	X 1C	-0.715	-0.750
X 2C	-0.707	-0.789	X 2C	-0.667	-0.702
Gal 1C	-0.020	0.008	Gal 1C	0.016	0.041
Gal 2C	0.030	0.041	Gal 2C	0.018	0.017
Gal 3C	-0.038	-0.002	H_2O 1C	-0.031	-0.042
H_2O 1C	-0.018	-0.037	H_2O 2C	-0.032	-0.029
H_2O 2C	-0.006	0.031			

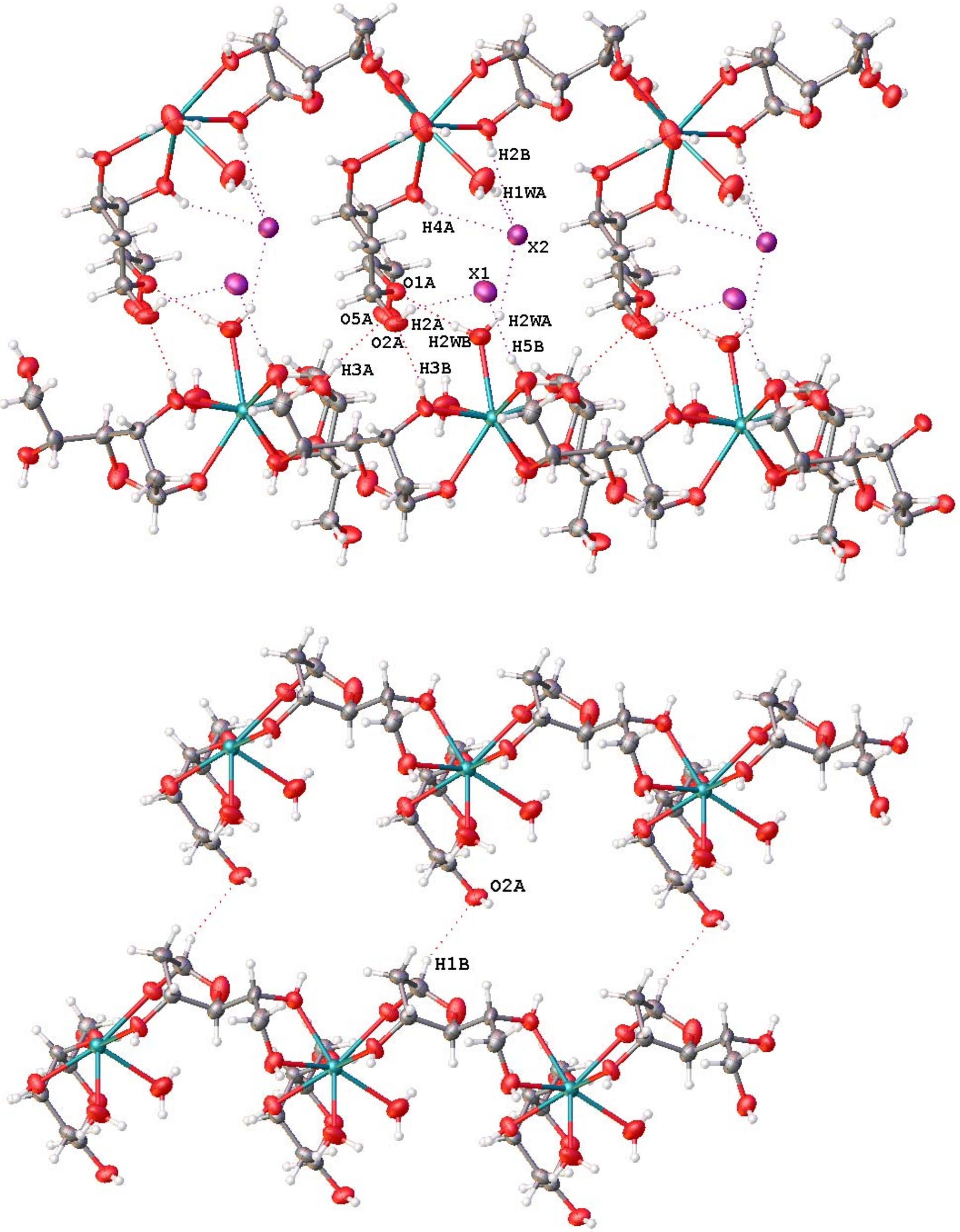

Figure S1. Intermolecular interactions connecting two next chains of the crystal structures. Bond lengths and angles are reported in Table S3.

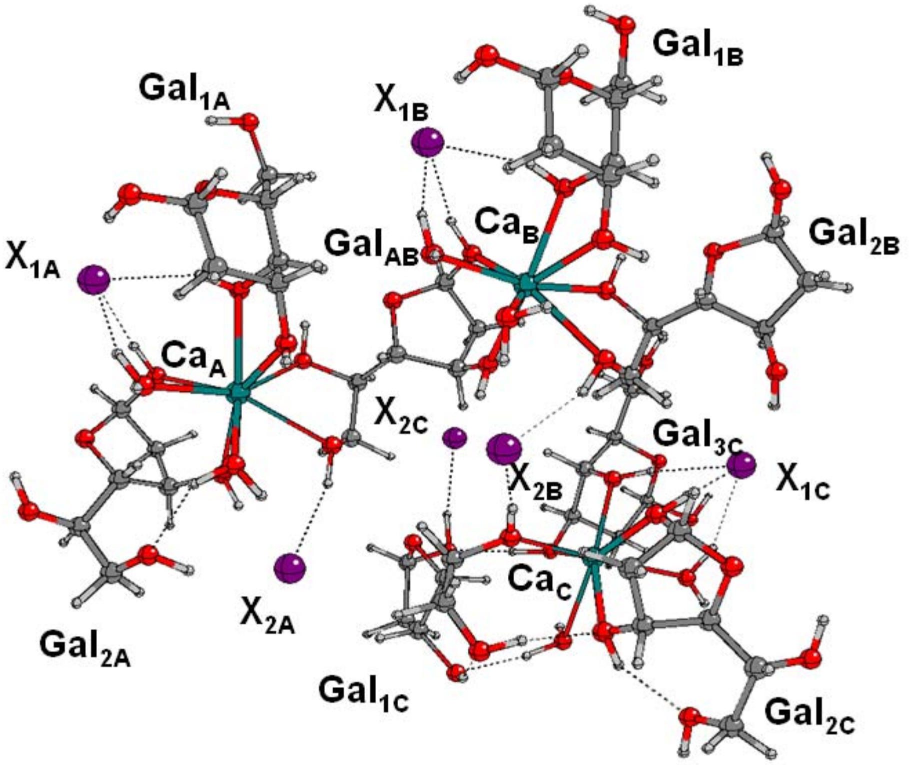

Figure S2: Labelling of groups of Table S4 for Fragment1.

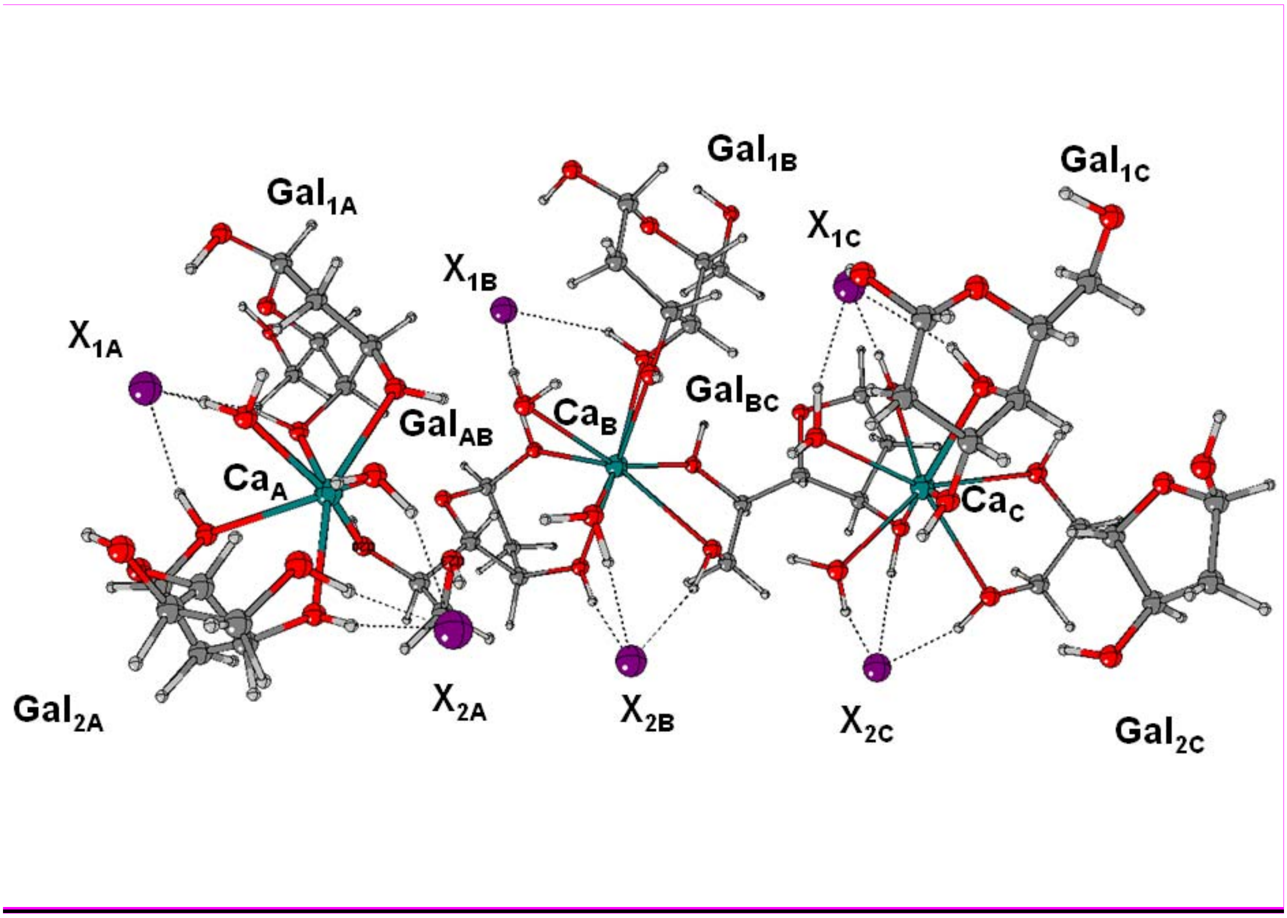

Figure S3: Labelling of groups of Table S4 for Fragment2.

Section S2. DFT simulations

S2.1 Computational details

The linear combination of gaussian-type function (LCGTF) approach as implemented in the CRYSTAL14 program has been used throughout. First, atomic coordinates coming from single crystal X-ray diffraction experiments were fully relaxed at fixed lattice parameters. At this stage, default thresholds[i] have been selected to control the level of numerical approximation in evaluating the Coulomb and exchange series (10^{-7} and 10^{-14}). The threshold on total energy changes has been set to 10^{-7} hartree between subsequent cycles in the geometry optimization procedure. RMS tolerances on gradients and atomic displacements of $3{\cdot}10^{-4}$ and $1.2{\cdot}10^{-3}$ respectively, have been applied. In subsequent self–consistent field (SCF) iterations, a 10^{-6} hartree threshold has been set for energy, while a 50% mixing of the Fock matrices and an eigenvalue level shift of 0.5 hartree have been exploited to accelerate convergence.[i] The reciprocal space has been sampled according to a regular sublattice defined by 4 points on each axis in the irreducible first Brillouin zone (IBZ). The exchange-correlation contribution to the total energy has been computed using the default pruned grid[i] for numerical integration, resulting in an average deviation for the electronic charge in the unit cell as low as $6{\cdot}10^{-3}$ *e* for CaDGalBr and $4{\cdot}10^{-3}$ *e* for CaDGalI.
Once convergence has been achieved, the coupled-perturbed (CP) Kohn-Sham method[ii–iv] as implemented in the CPKS module[i] of CRYSTAL14 has been exploited to extract from the Bloch-consistent periodic wavefunction information on optical axes, dielectric tensors and first- and second-order polarizabilities. To this end, thresholds on Coulomb and exchange series were lowered to either 10^{-14} or 10^{-28} hartree, while that on total SCF energy change has been reduced to 10^{-9} hartree. A finer grid in the Pack-Monkhorst net (keyword:[i] SHRINK/10 10) has been also selected. A Broyden scheme,[v] modified according to Johnson,[vi] with $W_0 = 10^{-4}$ and a 50 % mixing of the matrix second derivatives, has been applied to solve nonlinear SCF equations (keyword: BROYDEN/0.0001 50 2). The minimum allowed difference between non-degenerate unperturbed eigenvalues has been set to 10^{-6} hartree, in conjunction with a 10^{-3} threshold for the variation of the perturbation matrix elements $U^{\mathbf{k},tu}$ in subsequent coupled–perturbed iterations.
Previous results on similar sugar–based metal–organic frameworks[vii] showed that the CP-evaluated properties were reasonably converged with this set of parameters. Indeed, a considerably faster convergence against the BZ sampling and the number of terms in Coulomb and exchange series should be expected in large band gap systems than in the small band gap ones.[viii,ix] As concerns the present case, the band gap is roughly 7 eV, being as large as 7.56 eV and 6.91 eV in CaDGalBr and CaDGalI, respectively.

S2.2 Geometry optimization results

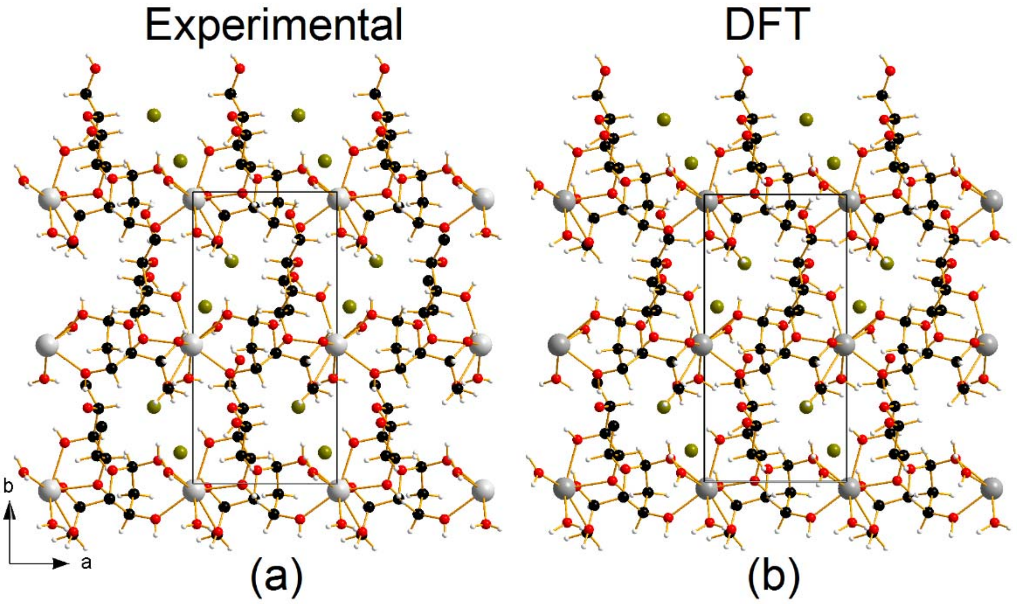

Figure S4. CaDGalBr experimental (a) and DFT PBE0–optimized (b) structures, as viewed along the *c* axis. Atoms are drawn in a ball–and–stick fashion with the following colour code: C black, H white, O red, Ca grey, Br dark green. The reference unit cell is also highlighted.

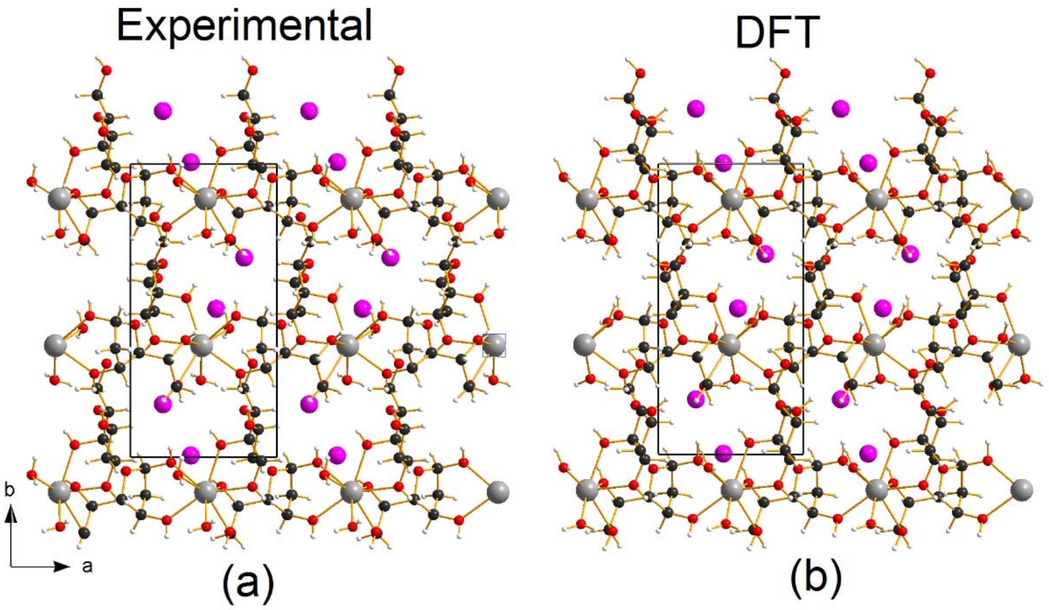

Figure S5. Same as Figure S1, for the CaDGalI experimental (a) and DFT PBE0–optimized (b) structure. Iodine ions are here drawn as purple spheres.

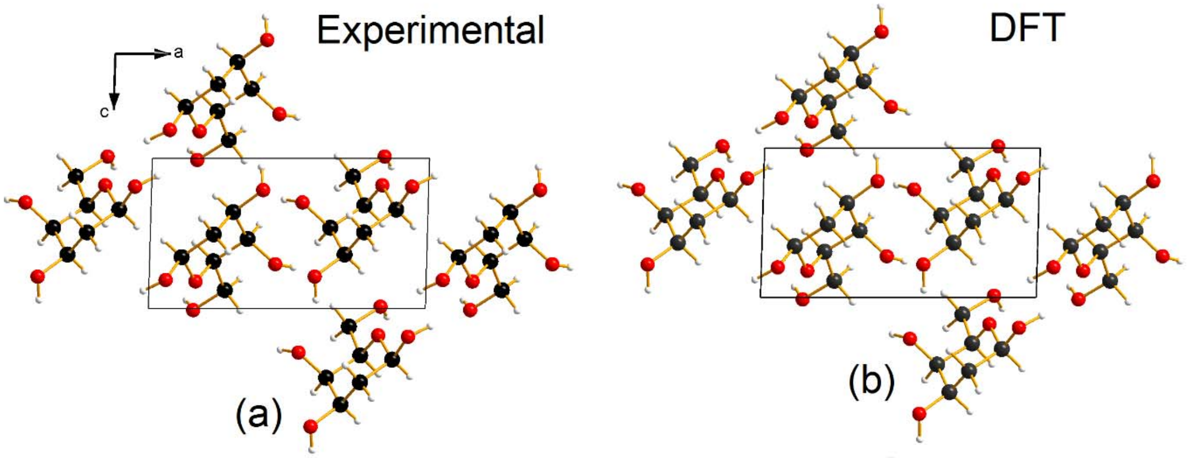

Figure S6. Same as Figure S1, for the 2-deoxy-D-galactose experimental (a) and DFT PBE0–optimized (b) structure, as seen down the *b* axis.

Table S5. Puckering parameters[x,xi] (*Q*: dimensionless amplitude; *θ*, *φ*: polar coordinates, degrees) for 2-deoxy-D-galactose conformers at the DFT–optimized geometries in CaDGalBr and CaDGalI, in comparison with those estimated from the experimental X–ray analysis.

Form	Structure	Method	*Q*	*θ* / deg	*φ* /deg	Label
Furanose	CaDGalBr	X-ray	0.3471	//	256.60	Envelope
		DFT	0.3446	//	249.28	Envelope
	CaDGalI	X-ray	0.3526	//	255.76	Envelope
		DFT	0.3471	//	256.60	Envelope
Pyranose	CaDGalBr	X-ray	0.5703	6.10	357.75	Chair
		DFT	0.5852	4.41	346.39	Chair
	CaDGalI	X-ray	0.5638	6.13	0.87	Chair
		DFT	0.6032	11.00	45.61	Distorted chair
	2-deoxy-D-galactose	X-ray	0.5938	2.31	212.14	Chair
		DFT	0.5933	2.06	226.03	Chair

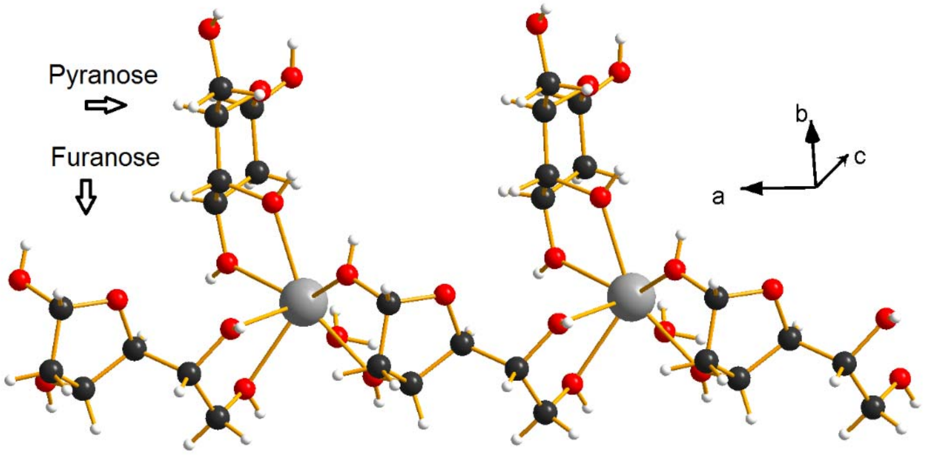

Figure S7. Zoom on the sugar: Ca^{2+} coordination mode in CaDGalBr and CaDGalI MOFs. Infinite Ca–furanose chains run approximately along the crystallographic *a* axis, while β–2–deoxy–D–galactopyranose extend in the free space along the *b* axis.

Comment:

DFT simulations are fully consistent with X-ray models. In most cases, the ring conformation of 2-deoxy-D-galactose (2d-Gal) is reproduced quantitatively (Figures S1–S3, Table S4). The only exception is the furanose form of 2d-Gal in CaDGalI, which results in a slightly distorted chair conformation upon DFT optimization. This is due to a slight rearrangement of the ring torsions, in turn prompted by a reorientation of the anomeric –OH to maximize the strength of hydrogen bonds with neighbouring furanose molecules. More in detail, the O1A–C2A–O2A–H2A torsion angle changes from –43.5 deg in the experimental structure to –87.3 deg in the DFT–optimized one (Figure 5). However, the associated distortion affecting the C, O sugar backbone is small on absolute grounds, the corresponding RMSD on the atomic positions being as low as 0.6 Å.

Table S6. DFT–optimized crystallographic coordinates of the asymmetric unit of 2-deoxy-D-galactose (DGal).

```
*******************************************************************************
LATTICE PARAMETERS (ANGSTROMS AND DEGREES) - BOHR = 0.5291772083 ANGSTROM
 PRIMITIVE CELL - CENTRING CODE 1/0 VOLUME=   362.429570 - DENSITY  1.503 g/cm^3
         A              B              C           ALPHA      BETA       GAMMA
     9.81100000     6.95300000     5.31500000    90.000000  91.580000  90.000000
 *******************************************************************************
 ATOMS IN THE ASYMMETRIC UNIT   23 - ATOMS IN THE UNIT CELL:   46
     ATOM              X/A                 Y/B                 Z/C
 *******************************************************************************
   1 T   8 O     5.965315172999E-02 -6.074025782232E-02 -2.023177850705E-01
   2 T   1 H    -2.181942650340E-02 -1.142487038391E-01 -1.202524616199E-01
   3 T   6 C     1.196609137393E-01 -2.028323248130E-01 -3.446059285494E-01
   4 T   1 H     4.373221080278E-02 -2.681597091485E-01 -4.759999649736E-01
   5 T   8 O     1.673029582642E-01 -3.500943103230E-01 -1.785749775778E-01
   6 T   6 C     2.365231702874E-01 -1.192013712857E-01 -4.908898459987E-01
   7 T   6 C     2.279139157373E-01  4.936672791947E-01 -3.068117723532E-01
   8 T   1 H     1.987594294960E-01 -9.324756024879E-03  3.771499719131E-01
   9 T   1 H     3.098668794602E-01 -5.354093422303E-02 -3.576566923369E-01
  10 T   6 C     3.052164658872E-01 -2.802510776124E-01  3.666793367430E-01
  11 T   1 H     1.539448227230E-01  4.328053209496E-01 -4.449925496127E-01
  12 T   6 C     2.661067558349E-01  3.414917160281E-01 -1.124476889199E-01
  13 T   6 C     3.529178016781E-01 -4.362875098681E-01 -4.482741561236E-01
  14 T   1 H     2.286008561754E-01 -3.451604968244E-01  2.390956359487E-01
  15 T   8 O     4.127239267139E-01 -2.067864970383E-01  2.214035608802E-01
  16 T   1 H     3.234938502697E-01  2.280347513492E-01 -2.051696675021E-01
  17 T   1 H     3.349122269399E-01  4.050436957353E-01  2.969320790387E-02
  18 T   8 O     1.530859480883E-01  2.642368519900E-01  1.249854839394E-02
  19 T   1 H     3.954767954219E-01  4.420803657750E-01  4.479847942574E-01
  20 T   8 O     4.539470507861E-01 -3.634446204376E-01 -2.764743313609E-01
  21 T   1 H     4.076281794906E-01 -2.609761053254E-01  5.167059531372E-02
  22 T   1 H     1.221408279417E-01  1.456664981151E-01 -7.240782281237E-02
  23 T   1 H    -4.825775367762E-01 -4.702778045513E-01 -2.449998191111E-01
```

Table S7. DFT–optimized crystallographic coordinates of the asymmetric unit of CaDGalBr.

```
*******************************************************************************
LATTICE PARAMETERS (ANGSTROMS AND DEGREES) - BOHR = 0.5291772083 ANGSTROM
 PRIMITIVE CELL - CENTRING CODE 1/0 VOLUME=  1049.033649 - DENSITY  1.779 g/cm^3
         A              B              C           ALPHA      BETA       GAMMA
     7.50347600    14.22876800    10.45196200    90.000000 109.936100  90.000000
 *******************************************************************************
 ATOMS IN THE ASYMMETRIC UNIT   55 - ATOMS IN THE UNIT CELL:  110
     ATOM                 X/A                 Y/B                 Z/C
 *******************************************************************************
   1 T 235 BR    9.057361478131E-02 -3.909856353767E-01 -2.590534264590E-01
   3 T 235 BR    2.832692337021E-01 -2.395069440442E-01  2.126194890246E-01
   5 T 220 CA   -1.898614135240E-02  4.765109667818E-01  2.486366932495E-01
   7 T   8 O    -2.642276626215E-01 -2.397405462063E-01 -4.757740259109E-02
   9 T   8 O    -4.471615564007E-01 -4.558184697829E-01  4.412487012965E-01
  11 T   8 O     1.622544241337E-01 -4.693630373385E-01  1.092914043468E-01
  13 T   1 H     2.341022220707E-01 -4.116432351990E-01  1.211183525935E-01
  15 T   1 H     1.989130274202E-01  4.904455922495E-01  4.748799961916E-02
  17 T   8 O    -2.993737789541E-01 -2.946590506909E-01 -2.594271312945E-01
  19 T   1 H    -1.826048164900E-01 -3.308670018999E-01 -2.452015834415E-01
  21 T   8 O     2.353846595643E-01 -4.200375446500E-01  4.023835421920E-01
  23 T   1 H     2.511072883013E-01 -3.610703600738E-01  3.599000242714E-01
  25 T   8 O    -3.870513532905E-02  3.580458963274E-01  7.813926823773E-02
  27 T   1 H    -1.398756752440E-01  3.398027718073E-01 -4.476094043944E-03
  29 T   1 H     7.133820830652E-02  3.253470863738E-01  7.103548246470E-02
  31 T   8 O    -3.546806859620E-01 -4.964789983428E-01  9.702627150886E-02
  33 T   1 H    -4.526131115870E-01  4.599159124493E-01  4.439612496587E-02
  35 T   8 O     2.520779666139E-01  3.745631296758E-01  3.574192118657E-01
  37 T   1 H     2.655268578921E-01  3.145599131808E-01  3.156616952433E-01
  39 T   8 O    -1.103056566432E-01 -3.533502840416E-01  1.985287842308E-01
  41 T   1 H    -3.246164294106E-02 -3.172545631095E-01  1.584704597154E-01
  43 T   8 O    -1.024308941287E-01  4.844798912929E-01  4.541554551988E-01
  45 T   1 H    -7.981562457363E-02 -4.641033277016E-01 -4.810008745880E-01
  47 T   8 O    -3.055514071956E-01 -6.989499766848E-02  7.800560114447E-02
  49 T   1 H    -2.479420457938E-01 -1.800504424414E-02  1.402308265476E-01
  51 T   8 O    -1.899899590664E-01  3.345039802695E-01  2.954945626132E-01
  53 T   1 H    -1.551074185818E-01  2.747679238908E-01  2.651392126689E-01
  55 T   6 C    -3.639340923120E-01 -3.050478053337E-01 -1.509678387383E-01
  57 T   1 H     4.856252487722E-01 -2.849886968710E-01 -1.875462028047E-01
  59 T   6 C     4.125756324961E-01 -4.429403412869E-01 -4.994671992705E-01
  61 T   1 H     4.605349464762E-01 -3.852019409525E-01 -4.268557729104E-01
  63 T   6 C    -3.358484240134E-01 -4.033719306013E-01 -9.187234603372E-02
  65 T   1 H    -1.851932061277E-01 -4.220745806092E-01 -6.098739058598E-02
  67 T   1 H    -4.169735257992E-01 -4.536029769134E-01 -1.688043680632E-01
  69 T   6 C     3.946561137196E-01  4.629892249271E-01 -4.371907632970E-01
  71 T   1 H     2.571681086064E-01  4.537871814917E-01 -4.247203800588E-01
  73 T   1 H    -4.922527060803E-01  4.547003259361E-01 -3.389022121326E-01
  75 T   6 C    -4.001551598268E-01 -4.079027261043E-01  3.132606555595E-02
  77 T   1 H     4.461086170882E-01 -3.959592079725E-01 -1.722105737775E-03
  79 T   6 C     4.270250834894E-01  3.935687739687E-01  4.623449396724E-01
  81 T   1 H     4.872258699367E-01  3.277359748971E-01 -4.868990125534E-01
  83 T   6 C    -3.055479009075E-01 -3.317423525912E-01  1.346308157580E-01
  85 T   1 H    -3.700877938265E-01 -3.309666681404E-01  2.141115424394E-01
  87 T   6 C    -4.330541295476E-01  4.471491503017E-01  4.101453176205E-01
  89 T   1 H    -4.718627767671E-01  4.392543033718E-01  2.998076554090E-01
  91 T   6 C    -3.381576930889E-01 -2.370654513239E-01  6.113302853408E-02
  93 T   1 H    -4.923726025111E-01 -2.253750420951E-01  1.961115910336E-02
  95 T   6 C    -2.264819383629E-01  4.176860510328E-01  4.811320353493E-01
  97 T   1 H    -1.979627534566E-01  4.134572366909E-01 -4.087409350270E-01
  99 T   6 C    -2.487165454411E-01 -1.551827966324E-01  1.522660971003E-01
 101 T   1 H    -2.983602394494E-01 -1.570517293601E-01  2.394506025245E-01
 103 T   1 H    -9.373019633945E-02 -1.613945298125E-01  1.888697886708E-01
 105 T   6 C    -1.784282608141E-01  3.236602262977E-01  4.332290510104E-01
 107 T   1 H    -3.447219977083E-02  3.032125992968E-01  4.968901761191E-01
 109 T   1 H    -2.753832342417E-01  2.688577044594E-01  4.434355009140E-01
```

Table S8. DFT–optimized crystallographic coordinates of the asymmetric unit of CaDGalI.

```
*******************************************************************************
 LATTICE PARAMETERS (ANGSTROMS AND DEGREES) - BOHR = 0.5291772083 ANGSTROM
 PRIMITIVE CELL - CENTRING CODE 1/0 VOLUME=  1121.076037 - DENSITY  1.949 g/cm^3
         A              B              C           ALPHA      BETA       GAMMA
     7.63840000    14.46030000    10.74940000    90.000000 109.228000  90.000000
 *******************************************************************************
 ATOMS IN THE ASYMMETRIC UNIT   55 - ATOMS IN THE UNIT CELL:  110
     ATOM                 X/A                 Y/B                 Z/C
 *******************************************************************************
   1 T 253 I     2.602466473769E-01  1.888498520419E-01  2.663050919937E-01
   3 T 253 I    -4.497548780645E-01 -4.981560136744E-01  2.397810860717E-01
   5 T 220 CA    4.905889956075E-01  3.761198257112E-01 -2.717069036608E-01
   7 T   8 O     1.564846882093E-01 -3.310418238509E-01  4.759735360385E-01
   9 T   8 O     3.436996138776E-02  4.492183279959E-01 -9.217625286002E-02
  11 T   8 O    -3.470960854892E-01  4.538155028435E-01 -4.069937266640E-01
  13 T   8 O     4.589212236975E-02 -3.531603099890E-01  2.535244918660E-01
  15 T   8 O    -2.747377931048E-01  4.779542436367E-01 -1.146344848826E-01
  17 T   8 O    -4.673244771783E-01  2.593884157830E-01 -4.199864844776E-01
  19 T   8 O     1.697391414897E-01  3.930368489301E-01 -4.314950115471E-01
  21 T   8 O    -2.387814303026E-01  2.775838414607E-01 -1.542387189788E-01
  23 T   8 O     3.706387663380E-01 -4.609586179784E-01 -3.078416607926E-01
  25 T   8 O     3.779874916830E-01  4.000493320886E-01 -8.555888075198E-02
  27 T   8 O     1.230512497447E-01 -1.894498144603E-01 -3.633390882751E-01
  29 T   8 O     3.161308042737E-01  2.349755308781E-01 -2.067507093330E-01
  31 T   6 C     4.823167885504E-02 -3.898910590249E-01  3.735456297321E-01
  33 T   6 C    -9.521889184253E-02  4.599214420466E-01 -2.574977643948E-02
  35 T   6 C     1.234729786970E-01 -4.870305593294E-01  4.001789946291E-01
  37 T   6 C    -9.870517597975E-02  3.680298554607E-01  4.086682128619E-02
  39 T   6 C     9.277165450490E-02  4.821186022382E-01 -4.724545386686E-01
  41 T   6 C    -6.607327821709E-02  2.991575052057E-01 -5.590716530410E-02
  43 T   6 C     1.749662306062E-01 -4.494204104177E-01 -3.590846632059E-01
  45 T   6 C     6.432048584501E-02  3.535503650707E-01 -1.123655965593E-01
  47 T   6 C     1.165064836191E-01 -3.506087620404E-01 -4.064192848589E-01
  49 T   6 C     2.714517956938E-01  3.342882362304E-01 -4.261160286928E-02
  51 T   6 C     2.018881343710E-01 -2.749561914802E-01 -3.079075572884E-01
  53 T   6 C     3.391601040614E-01  2.402466618177E-01 -6.903630541664E-02
  55 T   1 H    -2.958085840272E-01 -4.850139381558E-01 -3.818912264989E-01
  57 T   1 H    -3.873384384113E-01  4.531295954754E-01  4.973990367296E-01
  59 T   1 H    -5.807088765935E-02 -3.104764863516E-01  2.312797151583E-01
  61 T   1 H    -2.758096202856E-01 -4.589699559635E-01 -1.468987362503E-01
  63 T   1 H    -3.523840504375E-01  2.420122913177E-01 -4.339181238495E-01
  65 T   1 H     4.377075125383E-01  2.401082704238E-01  4.992761610277E-01
  67 T   1 H     8.703702078877E-02  3.491875592418E-01 -4.922851238523E-01
  69 T   1 H    -2.191885601611E-01  2.229469907012E-01 -2.009894573377E-01
  71 T   1 H     4.315121684739E-01 -4.161221770637E-01 -3.468642658747E-01
  73 T   1 H     4.076014688410E-01  4.514443622248E-01 -2.418161118395E-02
  75 T   1 H     2.124044181408E-01 -1.407189868830E-01 -3.261038753332E-01
  77 T   1 H     3.687083890528E-01  1.759007123445E-01 -2.217482227748E-01
  79 T   1 H    -9.446786434747E-02 -3.894558291910E-01  3.766754357012E-01
  81 T   1 H    -4.942916594369E-02 -4.816819496437E-01  4.196836056166E-02
  83 T   1 H     2.705925559103E-01 -4.882453268576E-01  4.096995963016E-01
  85 T   1 H     4.901116392240E-02  4.667396317947E-01  3.197287996415E-01
  87 T   1 H    -2.292006448880E-01  3.566428759080E-01  5.932181485790E-02
  89 T   1 H     1.556883435849E-02  3.638174489950E-01  1.341922721406E-01
  91 T   1 H    -5.712093817163E-02  4.790888202784E-01 -4.897295412699E-01
  93 T   1 H    -2.716050471166E-03  2.354859223581E-01 -6.062358136902E-03
  95 T   1 H     1.222747608385E-01 -4.662224075205E-01 -2.784180787225E-01
  97 T   1 H     2.883627577524E-02  3.419528197619E-01 -2.186890657174E-01
  99 T   1 H    -3.450342341857E-02 -3.467488349237E-01 -4.280860643434E-01
 101 T   1 H     3.019390404828E-01  3.408422663342E-01  6.414556641130E-02
 103 T   1 H     1.704565867301E-01 -2.900013266888E-01 -2.173516218355E-01
 105 T   1 H     3.527384280786E-01 -2.736457089308E-01 -2.848319585979E-01
 107 T   1 H     4.861370522530E-01  2.335225421170E-01 -1.045231720955E-02
 109 T   1 H     2.645567379647E-01  1.841189917032E-01 -3.966888465563E-02
```

REFERENCES

[i] Dovesi, R.; Saunders, V. R.; Roetti, C.; Orlando, R.; Zicovich-Wilson, C. M.; Pascale, F.; Civalleri, B.; Doll, K.; Harrison, N. M.; Bush, I. J.; D'Arco, P.; Llunell, M.; Causà, M.; Noël, Y. CRYSTAL14 User's Manual. University of Torino: Torino, **2014**

[ii] Ferrero, M.; Rerat, M.; Orlando, R.; Dovesi, R. *J. Comput. Chem*. **2008**, 29, 1450-1459.

[iii] Ferrero, M.; Rerat, M.; Orlando, R.; Dovesi, R. *J. Chem. Phys*. **2008**, 128, 014110.

[iv] Ferrero, M.; Rerat, M.; Kirtman, B.; Dovesi, R. J. *Chem. Phys*. **2008**, 129, 244110.

[v] Broyden, C. G. *Math. Comput*. **1965**, 19, 577-593.

[vi] Johnson, D. D. *Phys. Rev* B, **1988**, 38, 12807-12813.

[vii] Marabello, D.; Antoniotti, P.; Benzi, P.; Cariati, E.; Lo Presti, L.; Canepa, C. *Acta Cryst.* **2019**, B75(2), 210-218.

[viii] Lacivita, V.; Rérat, M.; Orlando, R.; Dovesi, R.; D'Arco, P. *Theor. Chem. A*cc. **2016**, 135, 81.

[ix] Lacivita, V.; Rérat, M.; Orlando, R.; Ferrero, M.; Dovesi, R. *J. Chem. Phys*. **2012**, 136, 114101.

[x] Cremer, D.; Pople, J. A. *J. Amer. Chem. Soc*., **1975**, 97,1354-1358

[xi] Boeyens, J. C. A. *J. Cryst. Mol. Struct.* **1978**, 8, 317-320.
